# Group A *Streptococcus* Prevents Mast Cell Degranulation to Promote Extracellular Trap Formation

**DOI:** 10.3389/fimmu.2018.00327

**Published:** 2018-02-27

**Authors:** Mary Clark, Jessica Kim, Neelou Etesami, Jacqueline Shimamoto, Ryan V. Whalen, Gary Martin, Cheryl Y. M. Okumura

**Affiliations:** ^1^Department of Biology, Occidental College, Los Angeles, CA, United States

**Keywords:** group A *Streptococcus*, mast cell, cathelicidin, granules, extracellular traps, cathepsin G

## Abstract

The resurgence of Group A *Streptococcus* (GAS) infections in the past two decades has been a rising major public health concern. Due to a large number of GAS infections occurring in the skin, mast cells (MCs), innate immune cells known to localize to the dermis, could play an important role in controlling infection. MCs can exert their antimicrobial activities either early during infection, by degranulation and release of antimicrobial proteases and the cathelicidin-derived antimicrobial peptide LL-37, or by forming antibacterial MC extracellular traps (MCETs) in later stages of infection. We demonstrate that MCs do not directly degranulate in response to GAS, reducing their ability to control bacterial growth in early stages of infection. However, MC granule components are highly cytotoxic to GAS due to the pore-forming activity of LL-37, while MC granule proteases do not significantly affect GAS viability. We therefore confirmed the importance of MCETs by demonstrating their capacity to reduce GAS survival. The data therefore suggests that LL-37 from MC granules become embedded in MCETs, and are the primary effector molecule by which MCs control GAS infection. Our work underscores the importance of a non-traditional immune effector cell, utilizing a non-conventional mechanism, in the defense against an important human pathogen.

## Introduction

Group A *Streptococcus* (GAS) causes a diverse spectrum of clinical manifestations in humans. In the skin, infections range from mild diseases, such as impetigo at the outer keratin layer, to severe invasive diseases, such as necrotizing fasciitis in the deep fascia (1). Strikingly, GAS is conservatively estimated to be responsible for over 140 million cases of impetigo globally each year (2). In particular, the globally disseminated serotype M1T1 subclone of GAS (M1 GAS) has been the most frequently isolated serotype from patients with either non-invasive or invasive disease (3). An important consequence of GAS infection is the increased propensity for developing post-streptococcal diseases, such as rheumatic heart disease (RHD). RHD poses a substantial worldwide health burden, resulting in significant morbidity and mortality (2, 4).

Because skin infections arise from streptococcal colonization of unbroken skin (5), cells in the epidermal layers are important first responders to GAS infection. Although typically associated with allergic inflammation, mast cells (MCs) are also found in some quantity at sites of interface with the environment, including skin, lung, and gut, and are well-positioned to control bacterial and viral infections (6, 7). MCs are hematopoietic cells characterized by their high content of secretory granules containing pre-formed mediators, including proteases, histamine, cytokines, and cathelicidin-derived antimicrobial peptide [LL-37 (8)]. Although the ability of MCs to release cytokines and recruit neutrophils to the site of infection is important (6, 8), it is becoming clear that MCs play a more direct role in combatting pathogenic bacteria, such as GAS; mice deficient in MCs are more susceptible to subcutaneous GAS infection (6). Release of granule compounds into the extracellular milieu is one potential mechanism by which MCs could exert antibacterial activity. Several MC proteases have direct antibacterial activity (9, 10) and LL-37 is an important host antibacterial molecule. Recently, it has also been shown that MCs form extracellular traps (ETs) to kill pathogens, including GAS. Similar to neutrophil extracellular traps (11), MCET formation is a controlled process, where nuclear DNA mixes with granule components, such as tryptase and LL-37, and released to form extracellular structures that ensnare and kill bacteria (12). Mast cell cultures (HMC-1) can directly kill various types of bacteria, including M23 serotype GAS, in an MCET-dependent manner (13). However, the extent to which MCETs contribute to bacterial clearance is unclear, as MCET formation results in the death of the normally long-living MCs, and M1 GAS has several mechanisms to avoid ET-mediated killing (14, 15).

An important antimicrobial component of ETs is LL-37. LL-37 is an amphipathic peptide that has been proposed to work on bacterial membranes by forming pores, compromising the transmembrane potential, and allowing cytoplasmic leakage (16). LL-37 is also significant for the formation and antimicrobial activity of ETs of both neutrophils and MCs (11, 13, 17). GAS is particularly susceptible to LL-37 (18), despite the fact that it has several virulence factors, such as the Scl-1 and M proteins, that confer LL-37 resistance (15, 19). MCs from mice that are deficient in the mouse cathelicidin-derived CRAMP have a reduced ability to control GAS infection (6). However, whether the effectiveness of CRAMP is due to direct release from granules or its activity in MCETs remains unclear.

Because the skin is constantly exposed to both environmental and resident microbes, the MC response to bacteria needs to be carefully managed to avoid inappropriate hyperinflammation, which could result in autoimmunity or tissue damage. MCET formation could, therefore, be a means by which bacterial infection could be controlled without negatively impacting the host. In this study, we sought to characterize the early MC response to M1 GAS to determine the importance of MC degranulation in bacterial clearance. We found that despite potent antibacterial activity, MC granules are not released in response to GAS. Furthermore, our data confirm the importance of LL-37 for the antibacterial activity of MCs and suggest that MCETs are the main mechanism by which MCs control GAS infection.

## Materials and Methods

### Cell Culture

HMC-1 cells (13) were maintained in Iscove-modified Dulbecco medium (IMDM) supplemented with 10% fetal bovine serum (FBS), 1.2 mM α-thioglycerol, and 100 U/mL penicillin/100 μg/mL streptomycin. Male C57Bl/6 mice (12 weeks old, The Jackson Lab) were used to harvest bone marrow-derived mast cells (BMMCs). All animal use and procedures were approved by the Institutional Animal Care and Use Committee of Occidental College. Cells from mice were pooled and cultured as previously described (20). Cell differentiation was confirmed by immunofluorescence microscopy using a CD117 antibody (Santa Cruz Biotechnology) and toluidine blue staining. For co-culture assays with bacteria, cells were washed twice in PBS at 800 × *g* for 10 min. and resuspended in antibiotic-free IMDM supplemented with 2% FBS (incubation medium).

### Bacterial Strains

Wild-type (WT) GAS strain M1T1 5448 (M1 GAS) was originally isolated from a patient with necrotizing fasciitis and toxic shock syndrome (21). An allelic exchange mutant lacking the *scl-1* gene (Δscl) and the plasmid-complemented mutant (Δscl + pscl) was generated in a previous report (19). *Lactococcus lactis* NZ9000, *Staphylococcus aureus* Newman strain (ATCC 25904), and M6 GAS JRS4 (M6 GAS) (22) were also used throughout the study for comparisons. Bacterial strains were cultivated in Todd-Hewitt broth (THB) at 37°C and grown to log phase for all experiments. Where indicated, bacteria were treated with 50 μU cathepsin G (Sigma), 100 µg/mL lysozyme (Sigma), and/or 4 µM LL-37 peptide (AnaSpec) for 60 min at 37°C.

### Cell Killing Assays

HMC-1 cells were plated at a density of 5 × 10^5^ cells in a 48-well suspension culture plate in incubation medium. BMMCs were plated at a density of 2 × 10^5^ cells in a 96-well culture plate in incubation medium. Log-phase bacteria were added at an MOI of 1 and plates were centrifuged at 500 × *g* to synchronize bacterial contact with cells. Co-cultures were incubated for the indicated times at 37°C, 5% CO_2_. Cells were lysed in 0.0025% Triton X-100 and bacteria were enumerated on THB agar plates. Bacterial survival was calculated as the percentage of the initial inoculum. Where indicated, HMC-1 cells were degranulated with 20 µg/mL compound 48/80 (Sigma) and washed with PBS to remove released granules, or treated with 10 µg/mL pepstatin A (Sigma), 10 µM E-64 (Tocris Bioscience), or 1 mM phenylmethanesulfonyl fluoride (PMSF, Acros Organics) for 60 min prior to addition of bacteria.

### HMC-1 Cell Viability Assay

Cell viability was measured using the Cell Counting Kit-8 (Sigma). 2.5 × 10^5^ HMC-1 cells in incubation medium were incubated at 37°C/5% CO_2_ in 96-well plates with the indicated bacterial strains at an MOI of 0.1 for 1 h. CCK-8 colorimetric solution was added and the plate was incubated for an additional hour. Absorbance at 450 nm was measured and the viability of the cells was compared with the uninfected control.

### Conditioned Medium Killing Assays

2 × 10^6^ cells/mL HMC-1 cells in incubation medium were treated with 20 µg/mL compound 48/80 for 1 h to degranulate MCs and release granule components into the supernatant. Cells were centrifuged at 800 × *g* for 10 min., and the conditioned supernatant containing MC granule compounds was collected. Supernatant from untreated cells was collected as the control. 2 × 10^5^ cfu log-phase bacteria in THB were combined with the conditioned medium in a 96-well plate. Bacteria and conditioned medium were centrifuged at 500 × *g* for 5 min and incubated for the indicated times at 37°C. Bacteria were enumerated on THA plates and survival was calculated as described above. Where indicated, conditioned medium was treated with 10 µM E-64, 1 mM PMSF (Acros Organics), 50 µg/mL aprotinin (Sigma), or 1 mM 4-(2-aminoethyl) benzenesulfonyl fluoride hydrochloride (AEBSF, Sigma). Bacterial survival was compared to the survival of the inoculum.

### Granule Release Assays

Granule release was measured by detection of β-hexosaminidase in the cell culture supernatant as previously described (23). Briefly, 5 × 10^4^ cells in Tyrodes solution supplemented with 10 mM HEPES and 0.04% bovine serum albumin were added to 96-well plates. Cells were infected with the indicated bacterial strains at an MOI of 10 for 90 min at 37°C. After incubation, the plate was centrifuged to collect supernatants containing released granules and supernatants transferred to a new 96-well plate. Remaining cells were lysed with 0.05% Triton X-100 to assess granules remaining within the cells. Supernatants and lysates were incubated with 3.5 mg/mL 4-nitrophenyl N-acetyl-β-D-glucosaminide (PNAG, Sigma) in citrate buffer (200 mM citric acid and 100 mM Na_2_HPO_4_·7H_2_O, pH = 4.5) for 90 min. at 37°C. Reactions were stopped with 100 mM glycine (pH = 10.7) and plate absorbance was read at 405 nm.

### MCET Assays

To induce MCET formation, cells were plated at a density of 2 × 10^5^ cells in a 96-well culture plate in incubation medium containing nuclease-inactivated FBS (24) and 25 nM phorbol-12-myristate-13-acetate (PMA, Sigma) (13), and incubated for 4 h. To dismantle MCETs, stimulated cells were incubated with 50 mU/mL micrococcal nuclease (MNase, Sigma) and 50 mU/mL myeloperoxidase (MPO, Sigma) 1 h prior to infection (13). Log-phase bacteria were added at an MOI of 0.1, and plates were centrifuged at 500 × *g* to synchronize bacterial contact with MCETs. Cultures were incubated for 1 h at 37°C, 5% CO_2_. Cultures were triturated with 0.0025% Triton X-100 and bacteria were enumerated on THB agar plates. Because of the variability of MCET production, bacterial survival was calculated as the percentage of the medium control to pool data from three independent experiments.

### Immunofluorescence Microscopy

Conditioned medium-treated or control medium-treated bacteria were incubated on coverslips, fixed with 4% paraformaldehyde for 15 min, and blocked with 5% goat serum in PBS. Bacteria were probed with mouse anti-human LL-37/CAP-18 antibody (0.25 µg/mL, clone 3D11, Hycult Biotech) overnight at 4°C. Bacteria were washed and detected with goat anti-mouse Alexa Fluor 488 antibody (Invitrogen). 30 µM propidium iodide (Sigma) was included to detect membrane-permeable and dead bacteria. Coverslips were mounted with Prolong antifade reagent (Invitrogen) and imaged on an inverted Leica TCS SP5 confocal microscope using a 63×/1.40 oil objective with 2–3× digital zoom at calibrated magnifications and recorded with LAS AF software (Leica).

### Transmission Electron Microscopy (TEM)

Bacteria were fixed with 2% gluteraldehyde in 0.1 M cacodylate buffer (pH = 7.4) for 1 h at room temperature. Samples were washed and post-fixed for 1 h each at room temperature in 1% OsO_4_ in 0.1 M cacodylate buffer and 3% uranyl acetate. Samples were dehydrated with a graded series of ethanol (50, 75, 95, 95, 100, and 100%) for 30 min at each step. Samples were infiltrated and embedded in Spurr’s plastic (25). Ultrathin sections (approximately 70–80 nm) were cut with a glass knife, counterstained with lead citrate, and examined with a Zeiss EM109 TEM. Images were taken at calibrated magnifications using a line replica.

### Statistical Analysis

All experiments were performed at least three independent times, with each sample performed in triplicate. Data shown are the compilation of all experimental replicates, average ± SEM. Data were analyzed in GraphPad Prism (v. 7.0b, GraphPad Software, Inc.) using the D’Agostino and Pearson normality test, one-way ANOVA, and Sidak’s multiple comparisons test or unpaired *t-*test as indicated.

## Results

### MCs Do Not Effectively Control GAS Growth

**Figure 1:**
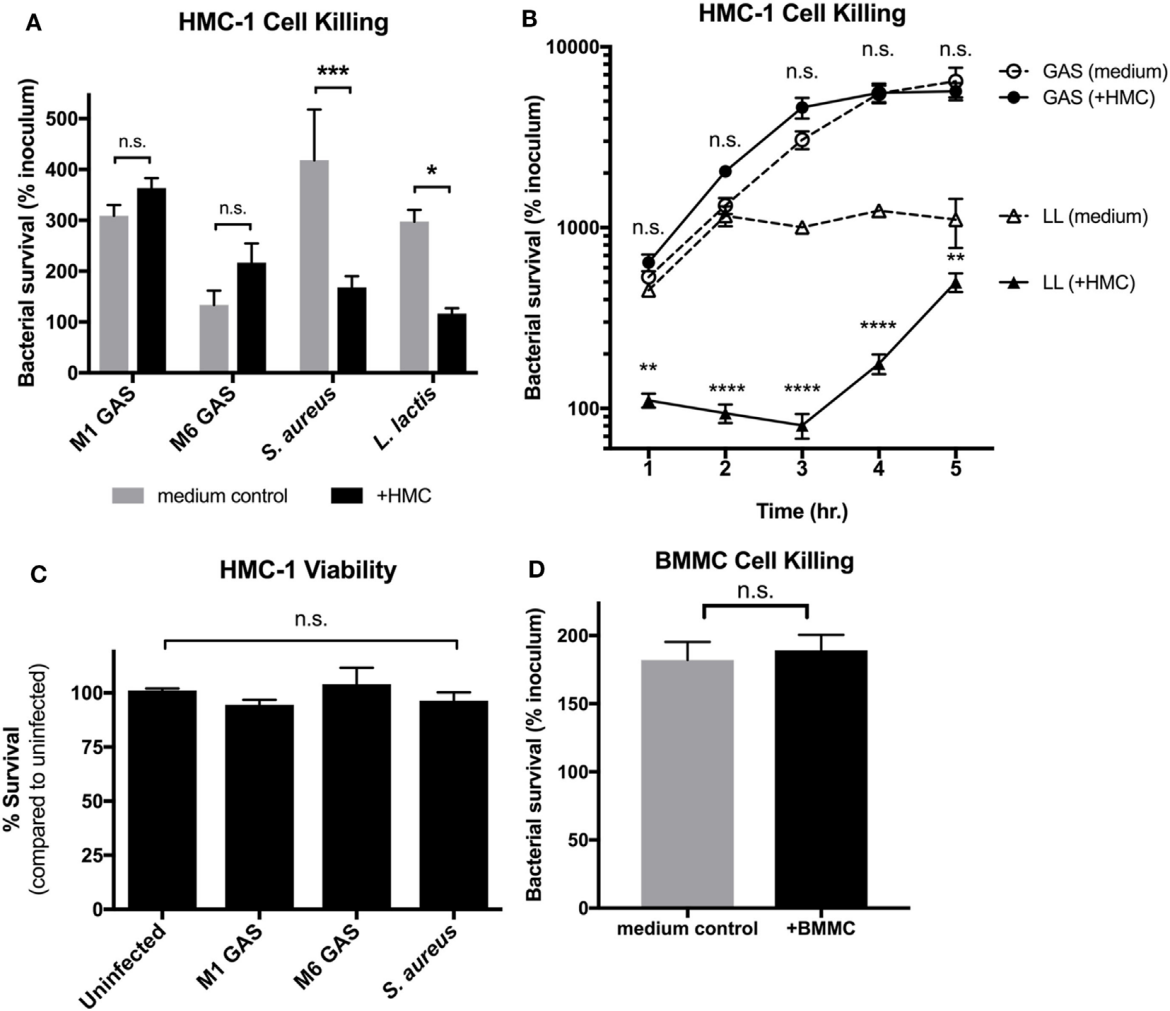
Mast cells (MCs) do not effectively control group A *Streptococcus* (GAS) growth. **(A)** Growth of the indicated strains in medium alone or with HMC-1 cells after 1 h of co-culture. Data are expressed as the percentage of the inoculum. **(B)** Growth of the indicated strains with HMC-1 cells compared with medium alone over time. Data are expressed as the percentage of the inoculum. **(C)** Viability of HMC-1 cells after incubation with indicated strains of bacteria. Data are compared to uninfected cells. **(D)** Growth of M1T1 subclone of GAS in medium alone or with BMMC after 1 h of co-culture. Data are expressed as the percentage of the inoculum. For all panels, data from at least three independent experiments were combined, and results are given in average ± SEM and analyzed by one-way ANOVA with Sidak’s multiple post-test [****P < 0.0001, ***P < 0.001, **P < 0.01, *P < 0.05, n.s., not significant; **(A–C)**], or by unpaired *t*-test **(D)**.

Since, MCET formation is a late-stage killing mechanism, MC degranulation and release of antimicrobial compounds could control GAS growth during early stages of infection. To determine whether MCs kill M1 GAS in early stages of infection, we co-cultured HMC-1 cells with M1 GAS and assessed bacterial survival at an early time point when MCETs would not yet be formed (13). Surprisingly, M1 GAS survived equally well in the presence or absence of HMC-1 cells after 1 h of co-culture (Figure 1A). An M6 serotype GAS strain also displayed similar survival trends in the presence of HMC-1 cells (Figure 1A). In contrast, HMC-1 cells effectively restricted the growth of other Gram-positive bacteria *Staphylococcus aureus* and *Lactococcus lactis* (Figure 1A). Accordingly, HMC-1 cells could not control the growth of M1 GAS at longer time points, whereas *L. lactis* growth continued to be restricted throughout the time course (Figure 1B). HMC-1 viability was not compromised when cells were incubated with any bacterial strain, including GAS, compared with uninfected cells (Figure 1C). HMC-1 cells represent a less mature MC due to a mutation in the co-stimulator c-kit and lack of the IgE receptor FcεRI (26, 27). To ensure that the inability to control GAS was not restricted to the HMC-1 cell line, we also tested primary BMMCs. Similarly to HMC-1 cells, BMMC could not restrict M1 GAS growth after 1 h of co-culture (Figure 1D). These data demonstrated that MCs do not effectively control GAS growth. Importantly, the failure of MCs to control bacterial growth in the early stage of infection suggests that MC degranulation could be impaired.

### Granule Release Is Necessary for Human MCs to Effectively Kill GAS

**Figure 2:**
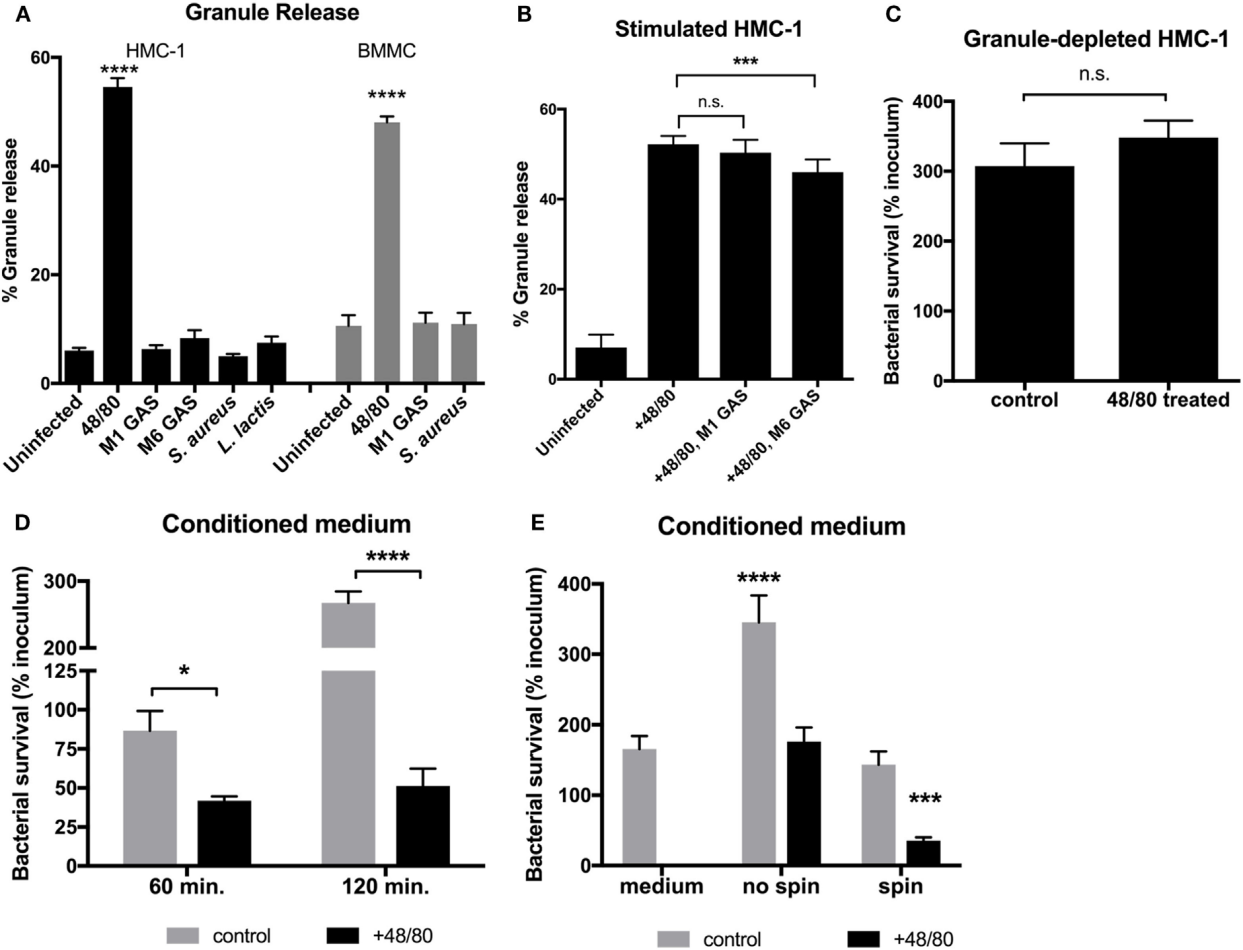
Granule release is necessary for MCs to effectively kill group A *Streptococcus* (GAS). **(A)** Beta-hexosaminidase granule release assay with HMC-1 cells (solid bars) or bone marrow-derived MC (shaded bars) incubated with the indicated strains. **(B)** Beta-hexosaminidase granule release assay with compound 48/80-treated HMC-1 cells incubated with the indicated strains. For statistical analysis, data were compared to granule release from uninfected cells **(A)** or 48/80-treated cells **(B)**. **(C)** GAS growth with untreated (control) or granule-depleted HMC-1 cells (cells treated with compound 48/80 and washed to remove released granules). **(D,E)** GAS survival with supernatant collected from untreated (control) or compound 48/80-treated HMC-1 cells (conditioned medium). In panel **(E)**, GAS and supernatants were centrifuged or not as indicated prior to incubation. For statistical analysis, data was compared to GAS grown in control supernatants **(D)** or medium alone **(E)**. For all panels, data from at least three independent experiments were combined, results are given in average ± SEM and analyzed by one-way ANOVA with Sidak’s multiple post-test (****P < 0.0001, ***P < 0.001, *P < 0.05, n.s., not significant).

Although MCs are capable of phagocytosing certain bacteria, GAS is not phagocytosed by MCs and are thus killed by extracellular mechanisms (13). One explanation for the inability of MCs to control GAS growth (Figure 1) is that MCs do not release their granules. We, therefore, sought to determine if MCs degranulate in response to GAS by measuring release of the granular enzyme β-hexosaminidase into the supernatant as a marker for degranulation. The results illustrate that while granules were released by both HMC-1 cells and BMMCs upon control treatment with the MC activator compound 48/80, infection with either M1 or M6 serotype GAS did not stimulate the release of granules compared with the uninfected control (Figure 2A). Interestingly, other Gram-positive bacteria also failed to stimulate granule release from MCs (Figure 2A). To explore the possibility that GAS-triggered degranulation requires co-stimulation, we next examined incubation of HMC-1 cells with compound 48/80 in combination with GAS. M1 GAS did not significantly enhance 48/80-mediated granule release from HMC-1 cells. Intriguingly, when co-incubated with M6 serotype GAS and compound 48/80, HMC-1 cells released fewer granules compared with HMC-1 cells treated with compound 48/80 alone (*p* < 0.001, Figure 2B). Thus, the data show that GAS infection does not promote and may even inhibit the release of granules from MCs. Corroborating this data, we found that M1 GAS survived equally well with untreated HMC-1 cells containing granules (control) or HMC-1 cells treated with compound 48/80 and depleted of granules prior to infection (Figure 2C). Together, these data suggest that MCs cannot effectively restrict GAS growth, either because degranulation is not stimulated or is actively inhibited by the bacteria.

To investigate whether HMC-1 granules contain active compounds that are effective against GAS, we incubated GAS with granule components released into the supernatant from compound 48/80-treated HMC-1 cells. We found that conditioned medium containing granule components potently killed M1 GAS as shown by a significant decrease in bacterial survival compared to untreated cell supernatant controls (Figure 2D). Thus, HMC-1 granules have antimicrobial components capable of killing GAS. Though we could observe an antimicrobial effect of the granule compounds in the conditioned medium when incubated with bacteria (Figure 2E, no spin), contact facilitated by centrifuging conditioned medium/bacteria co-cultures was required to reduce the number of GAS below the medium only control (Figure 2E, spin). Taken together with Figure 1, our data suggest that GAS does not stimulate or actively inhibits MC degranulation as a strategy to evade MC granule-mediated killing.

### Cathelicidin Found in MC Granules Is a Potent Anti-GAS Effector

**Figure 3:**
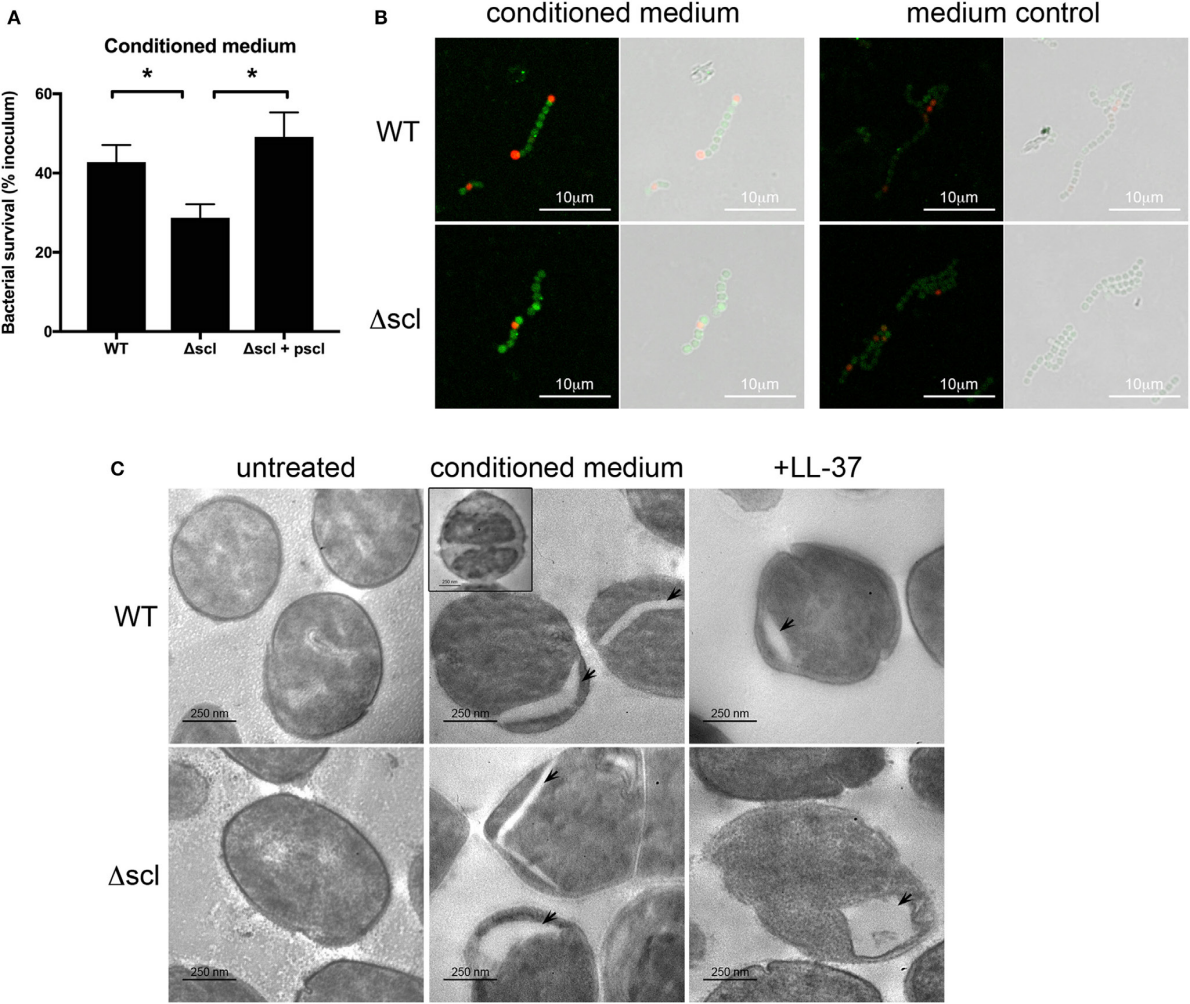
LL-37 in mast cell granules is a potent anti-group A *Streptococcus* effector. **(A)** Survival of WT, Δscl, and Δscl plasmid-complemented (Δscl + pscl) strains with HMC-1 conditioned medium. Data from at least three independent experiments were combined, results are given in average ± SEM and analyzed by one-way ANOVA with Sidak’s multiple post-test (**P < 0.01, *P < 0.05). **(B)** Merged immunofluorescence and corresponding phase contrast images of WT and Δscl strains incubated with or without HMC-1 conditioned medium. LL-37 is indicated in green, propidium iodide in red. Images taken at 63× magnification with 2.5× digital zoom. **(C)** Transmission electron microscopy images of WT and Δscl strains incubated with media (untreated), HMC-1 conditioned medium or LL-37 peptide. Arrows indicate empty cytoplasmic areas. Inset: bacterial cell with intact membrane and less electron-dense cytoplasm (ghost). Images taken at 20,000× magnification.

Mast cell granules, which are potentially cytotoxic to bacteria (Figure 2D), contain several proteases, cytokines, and antimicrobial peptides, including LL-37 (8, 12). Evidence from previous mouse studies has demonstrated a role for MC-derived cathelicidin in suppressing GAS infection in the skin (6), and thus LL-37 may be the major anti-GAS effector in MC granules. Cathelicidin potently kills GAS *in vitro, in vivo*, and in extracellular traps (13, 28). However, GAS has several virulence factors that limit the cytotoxic effects of LL-37, including the surface protein streptococcal collagen-like 1 protein [Scl-1 (19)]. GAS lacking Scl-1 is highly susceptible to LL-37 (19). To determine whether LL-37 in MC granules is responsible for the potent antibacterial activity of MC granules, we incubated HMC-1 conditioned medium with the LL-37-sensitive mutant M1 GAS lacking Scl-1 (Δscl) and assessed bacterial survival compared with wild type (WT) and the plasmid-complemented strain (Δscl + pscl). As expected, conditioned medium effectively killed all strains of GAS, but killed the Δscl GAS significantly better than either the WT or Δscl + pscl strains (Figure 3A). Thus, treating the Δscl strain with conditioned medium containing MC granule components effectively recapitulates the trend observed with LL-37 treatment alone (19).

To further confirm the antibacterial activity of LL-37 in MC granules, we next examined the effects of treating GAS with conditioned medium by microscopy. By immunofluorescence microscopy, we observed LL-37 on the surface of condition medium-treated bacteria (Figure 3B). No significant LL-37 staining was observed when bacteria were incubated in control supernatant from untreated HMC-1 cells (Figure 3B). Both WT and Δscl strains showed similar staining with the LL-37 antibody (Figure 3B). Interestingly, LL-37 binding did not necessarily correlate with membrane permeabilization, as indicated by a general lack of propidium iodide staining of LL-37-positive cells (Figure 3B).

We also observed the effects of granule-treated bacteria by TEM. We observed no differences in cell or membrane morphology between untreated WT or Δscl strains of bacteria (Figure 3C). When treated with conditioned medium containing MC granule components, we observed in both strains which appears to be either a separation of the bacterial membrane from the bacterial cytoplasm or empty areas in the cytoplasm (Figure 3C, arrowheads). Consistent with this observation, we also observed bacterial cell “ghosts,” where bacterial cells were less electron dense, but maintained an intact membrane (Figure 3C, WT strain inset). We also observed some cells that appeared to be swollen to approximately twice the size of untreated bacterial cells with a less electron dense cytoplasm (data not shown). The membrane separation and gaps in the cytoplasmic space observed with granule-treated bacteria were similar to the effect of treating the bacteria with LL-37 peptide alone (Figure 3C). Consistent with these observations, some intact LL-37-positive bacteria were permeable to propidium iodide, indicating a breach, but not a complete disruption in the bacterial membrane (Figure 3B). Though more cells with the gaps in the cytoplasmic space were observed for the LL-37-sensitive Δscl strain than the WT strain (Figure 3C), the differences were not quantitated because treatment with granule components in conditioned medium led to large amounts of bacterial death of both strains (Figure 3A) and dead and lysed bacteria were not retained in the sample when processed for TEM (Figure 3C and data not shown). Together, these data suggest that the major anti-GAS effector of MC granules is LL-37.

### MC Granule Proteases Are Not Effective against GAS

**Figure 4:**
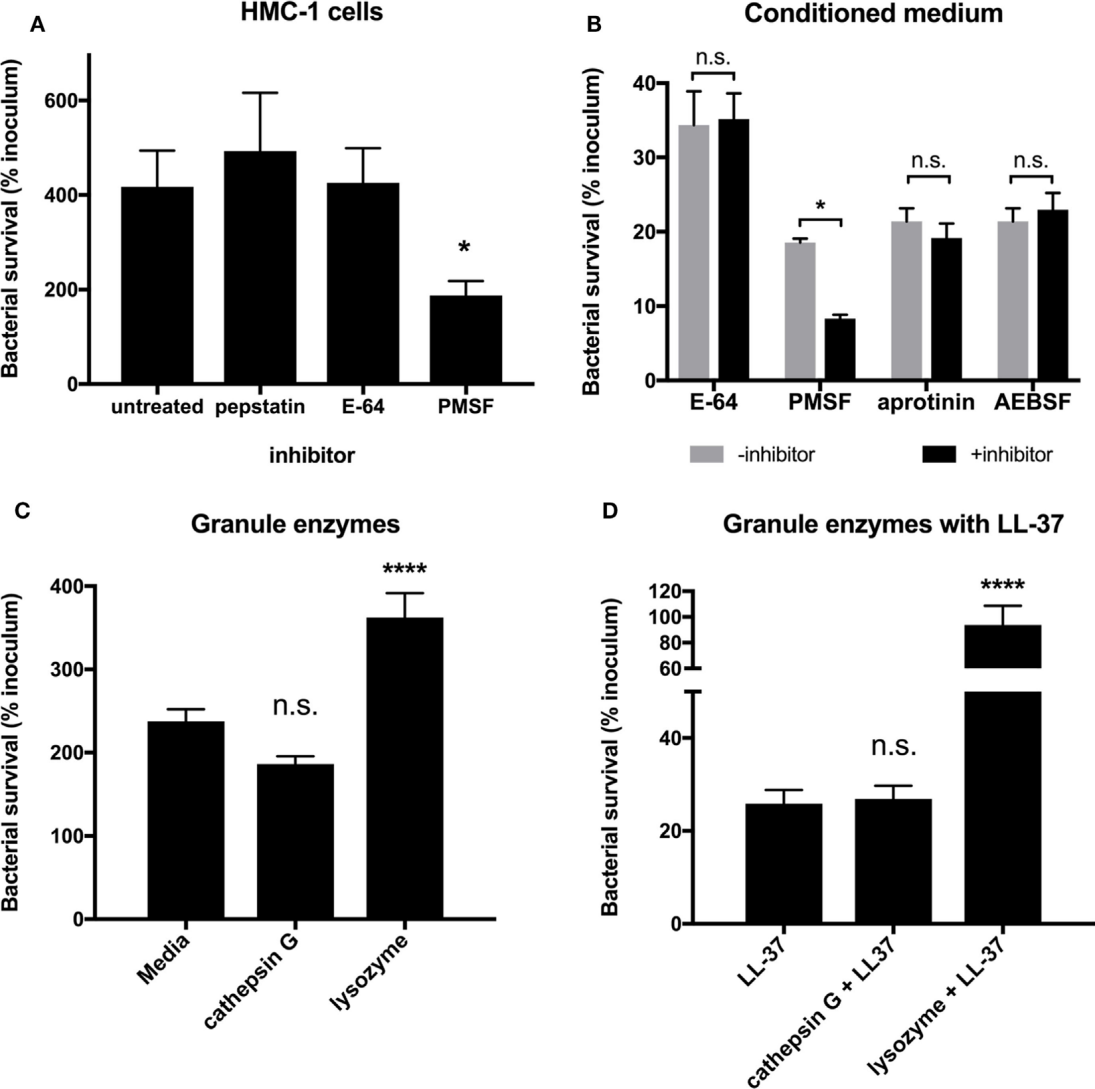
Mast cell granule proteases are not effective against group A *Streptococcus* (GAS). **(A)** Survival of GAS incubated with HMC-1 cells treated with the indicated protease inhibitor. For statistical analysis, data are compared with untreated cells. **(B)** Survival of GAS incubated with HMC-1 conditioned medium treated in the presence or absence of the indicated protease inhibitor. For statistical analysis, data are compared with their respective control. **(C)** Survival of GAS incubated with the indicated granule enzymes. For statistical analysis, data are compared with bacteria grown in media alone. **(D)** GAS survival incubated with LL-37 in combination with the indicated granule enzymes. For statistical analysis, data are compared with LL-37 treatment. Data from at least three independent experiments were combined, results are given in average ± SEM and analyzed by one-way ANOVA with Sidak’s multiple post-test (****P < 0.0001, *P < 0.05, n.s., not significant).

While LL-37 is a major MC granule effector (Figure 3), MC granules contain a myriad of proteases, some with the potential to directly kill bacteria (8, 9). To explore whether MC granule proteases are effective against GAS, we used a panel of protease inhibitors and determined their effects on the ability of MCs to kill GAS. When we treated HMC-1 cells with the aspartic acid protease inhibitor, pepstatin A and the cysteine protease inhibitor E-64, we observed no significant difference in the ability of HMC-1 cells to control the growth of M1 GAS compared with untreated cells (Figure 4A). Unexpectedly, when we treated HMC-1 cells with the serine protease inhibitor PMSF, we observed that the cells’ ability to limit M1 GAS growth was significantly improved (Figure 4A). This result contradicted our expectation that GAS would grow more abundantly in the presence of a protease inhibitor, as we hypothesized MC proteases exerted defense against GAS growth.

Because our previous data demonstrated that conditioned medium containing MC granule components had significant anti-GAS activity (Figures 2 and 3), we incubated conditioned medium with protease inhibitors to better determine whether granule proteases impact GAS. As with live HMC-1 cells, E-64 had no significant impact on the ability of conditioned medium to control GAS growth (Figure 4B). However, similar to live HMC-1 cells, PMSF enhanced the ability of conditioned medium to kill GAS (Figure 4B). To further investigate the potential role of granule proteases and probe the enhancing properties of PMSF, we also incubated isolated granules with two other serine protease inhibitors, aprotinin and AEBSF (Figure 4B). Aprotinin and AEBSF have been reported to inhibit both granule serine proteases, such as cathepsin G and elastase (9, 29), and kallikreins, enzymes that processes the full length cathelicidin protein hCAP18 to the active LL-37 peptide (30). Neither aprotinin nor AEBSF had an effect on the ability of the isolated granules to kill GAS (Figure 4B). Thus, granule proteases are not responsible for the anti-GAS activity which we observe with HMC-1 conditioned medium (Figure 2).

Although adding protease inhibitors to conditioned medium did not alter the ability of granules to kill M1 GAS (Figure 4B), it is possible that the protease inhibitors were not delivered at an effective concentration or location. Therefore, we asked whether purified MC granule proteases affected the survival of GAS. We chose to examine cathepsin G, a protease found in both MC and neutrophil granules with potential direct antibacterial activity (9, 10) and lysozyme, an enzyme that would not be inhibited by any of the protease inhibitors tested (Figure 4B). We could not determine a minimum inhibitory concentration for either cathepsin G (>25 μU/mL) or lysozyme (>5 mg/mL) for M1 GAS. When M1 GAS was incubated with purified cathepsin G or lysozyme for short time periods (Figure 4C), we observed no negative impact on GAS survival compared with bacteria in media alone (Figure 4C), and lysozyme significantly enhanced bacterial growth (Figure 4C). Because MC granules are complex mixtures of proteases, cytokines, and LL-37, it is possible that enzymes work in concert with antimicrobial peptide. For example, lysozyme could trim the outer peptidoglycan layer to allow LL-37 to more readily access the bacterial membrane. To test this, we incubated bacteria with both LL-37 and either cathepsin G or lysozyme. As seen previously, LL-37 alone had potent anti-GAS activity (Figure 4D). However, neither enzyme enhanced the ability of LL-37 to kill GAS (Figure 4D). The increased survival of GAS with lysozyme and LL-37 compared to LL-37 alone is likely due to the enhancement of bacterial growth by lysozyme that must be overcome by the LL-37 (Figures 4C,D). Taken together with our protease inhibitor data, our findings suggest that proteases found in MC granules are unlikely to be involved in the direct killing of GAS by MC granules, further supporting the idea that LL-37 is the sole anti-GAS mediator of MC granules.

### MCETs Control GAS Growth

Altogether, our data suggest that MCs fail to control GAS growth in early phases of infection due to the inability to degranulate (Figures 1 and 2). LL-37 in MC granules has potent anti-GAS activity (Figure 3), suggesting that MCET killing may be the primary mechanism by which MCs control GAS growth. However, M1 GAS encodes DNase and M1 protein that allow GAS to avoid MCET-mediated killing (14, 15). These proteins, in addition to the rapid growth of M1 GAS, may obscure any effect of MCETs that are produced at later time points during infection (Figure 1B). To directly test whether MCETs could control M1 GAS growth, we induced MCET formation with PMA and incubated the resulting MCETs with M1 GAS. We found that M1 GAS grew less well in the presence of both HMC-1 and BMMC MCETs compared with MCETs treated with MNase, an exonuclease used to dismantle the DNA backbone, and MPO to degrade tryptase (Figures 4A,B) (13). These data confirmed that intact MCET structures were required for the observed GAS growth restriction, and support that MCs control GAS infection by forming MCETs.

## Discussion

**Figure 5:**
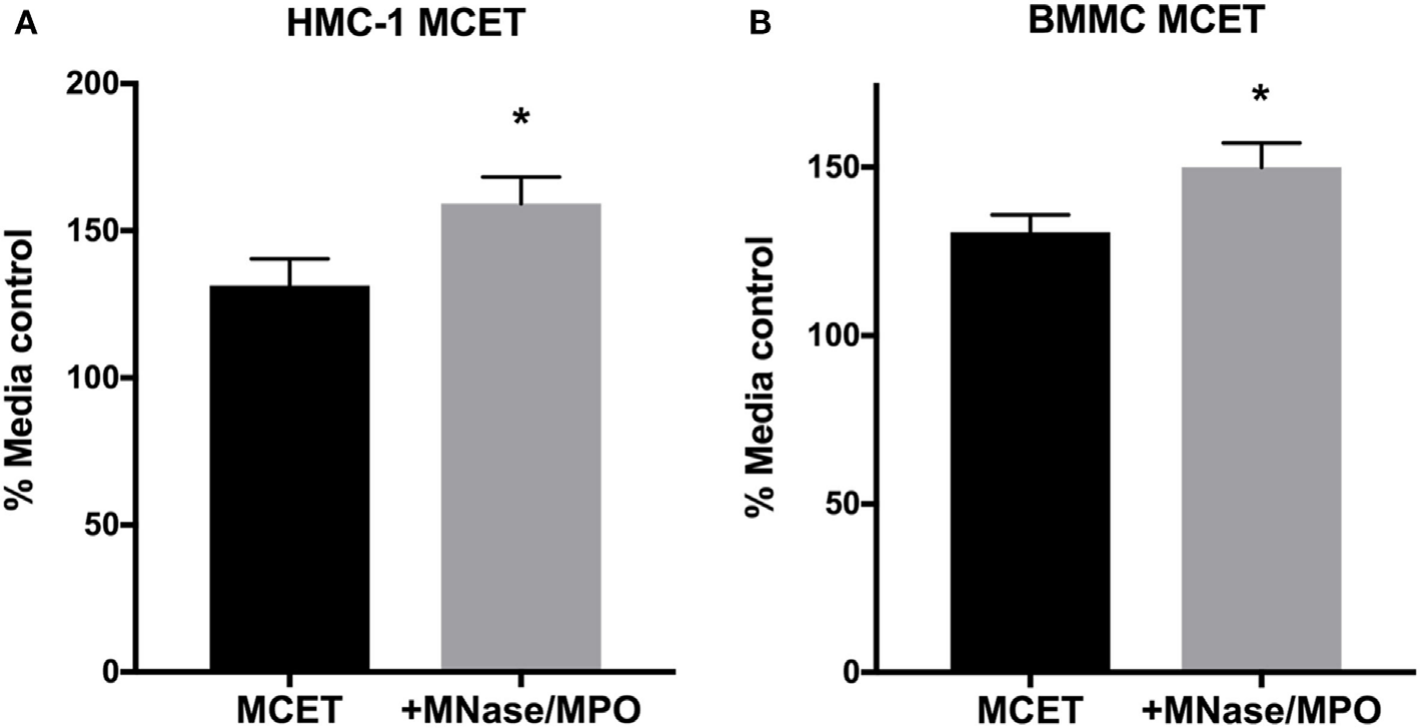
MC extracellular traps (MCETs) control group A *Streptococcus* (GAS) growth. Survival of GAS incubated for 1 h with HMC-1 **(A)** or bone marrow-derived mast cell MCETs **(B)**. As a control, MCETs were incubated with MNase and myeloperoxidase to dismantle MCETs. Data from at least three independent experiments were combined, results are given in average ± SEM and analyzed by one-way ANOVA with Sidak’s multiple post-test (*P < 0.05).

Group A *Streptococcus* is a major cause of skin and soft tissue infections worldwide. The diverse clinical manifestations of GAS infection suggest that innate immune cells, including MCs, are crucial to the control of GAS and may play a role in infection outcome. MCs are unable to control GAS in early stages of infection and do not release granules in response to GAS (Figures 1 and 2). We propose that MCs control GAS infection primarily through LL-37 found in MCETs (Figures 3 and 5). These findings indicate that although GAS prevents MC granule release, MCs are still able to control GAS infections through formation of MCETs. Our results are in concordance with previous studies, which found that bacterial growth is not effectively controlled until later time points when MCETs are more likely to form (6, 13). Moreover, this work confirms that LL-37 is the primary anti-GAS effector in MCs (6).

Extracellular bacterial killing by MCs is usually attributed to the release of granules into the extracellular milieu (8). This strategy may be particularly important for MC control of GAS infection, since MCs do not phagocytose GAS (13). We found that conditioned medium containing MC granule components potently killed GAS, yet granules were not released in response to GAS infection (Figure 2). HMC-1 cells are indeed capable of degranulating in response to live pathogens (31, 32) as well as certain bacterial products (33, 34), and express pattern recognition receptors responsible for detecting Gram-positive bacteria (26, 35, 36). Certain GAS proteins, such as SpeB and streptolysin O, can stimulate MC degranulation in high concentrations (20, 33, 37), but their relatively low concentrations in log phase bacterial culture supernatant indicates that these proteins likely do not contribute to MC degranulation in the context of an infection (38, 39). Similarly, other studies have found MCs fail to degranulate in response to Gram-positive bacterial products (40–42). Together, these results suggest more broadly that MC degranulation may require other stimulation that may be present in the context of an infection, such as skin beta defensins or skin microbiota (7, 43).

Limiting MC degranulation may initially be advantageous to GAS, as we confirmed that GAS is potently killed by the antimicrobial peptide LL-37 of MC granules [Figure 3 (6, 13)]. Our data and data from previous studies suggest that Gram-positive bacteria, such as GAS, have mechanisms to prevent or repress degranulation of MCs (Figure 2B) (39, 44). However, preventing degranulation and retaining LL-37 may in fact promote better MCET formation, since LL-37 contributes to nuclear degradation (17). Retaining LL-37 in MCETs may also be important to maintain sufficient concentrations of LL-37, as we found that contact/close proximity between conditioned medium containing granule components and GAS was required for bactericidal activity (Figure 2E). GAS is a potent inducer of MCETs (13) and bacterial products, such as M1 protein, promote MCET formation (15). Other studies also suggest an inverse relationship between degranulation and MCET formation. MCs form reduced amounts of MCETs in response to *Enterococcus faecalis* and *Candida albicans* compared with GAS, perhaps due to the fact that they also degranulate in response to these pathogens (31, 45).

LL-37 is an amphipathic peptide that has been proposed to exert its antimicrobial effect through toroidal pore formation in bacterial membranes (16). While membrane blebbing has previously been observed for GAS treated with high concentrations of LL-37 (46), specific morphology of bacteria undergoing LL-37-induced lysis has been difficult to capture by TEM, as lysed bacteria are not easily retained during sample processing. When we exposed GAS to either conditioned media from HMC-1 cells or synthetic LL-37, we observed a separation of the membrane and/or cell wall from the cytoplasm and large areas of empty cytoplasm by TEM (Figure 3C). This observation suggests that there are changes in GAS membrane permeability due to cytoplasmic leakage, rather than the creation of large pores that result in GAS lysis (47, 48). Indeed, recent studies have provided evidence that LL-37 toroidal pores are smaller than the critical hole size required for bacterial lysis (>9 vs. 15–24 nm, respectively) (16, 47). We and others observed that LL-37 does not cause significant membrane damage to GAS [Figure 3B (49)], suggesting that MC-derived LL-37 may create small pores that result in a change in membrane permeability, leading to an influx of water that eventually leads to bacterial cell lysis.

We propose a model of infection in which GAS prevents MC degranulation, which triggers the formation of MCETs. LL-37 is deposited in MCETs and subsequently kills GAS by affecting membrane integrity. Altogether, our work underscores the importance of LL-37 in MC granules as a potent effector against GAS infections and suggests that MCETs may play a more prominent role in the control of GAS infections than degranulation. M1 and other serotypes of GAS have virulence factors, such as DNase and M1 protein, that allow GAS to avoid MCET-mediated killing (14, 15). Such proteins are, therefore, attractive targets for therapy, as they are non-essential bacterial proteins whose inhibition would greatly enhance bacterial clearance by host innate immune cells, thus avoiding the development of drug resistance.

## Ethics Statement

This study was carried out in accordance with the recommendations of the Institutional Animal Care and Use Committee of Occidental College. The protocol was approved by the Institutional Animal Care and Use Committee of Occidental College.

## Author Contributions

MC and CO designed the experiments. MC, JK, NE, JS, RW, and CO performed research and analyzed and interpreted data. GM analyzed and interpreted EM experiments and contributed to the manuscript. MC and CO wrote the manuscript.

## Conflict of Interest Statement

The authors declare that the research was conducted in the absence of any commercial or financial relationships that could be construed as a potential conflict of interest.

## Abbreviations

GAS, group A *Streptococcus*; MC, mast cell; MCET, mast cell extracellular trap; ET, extracellular trap; M1 GAS, M1T1 subclone of GAS; MNase, micrococcal nuclease; MPO, myeloperoxidase.
